# Spatial and Temporal Analysis of Dengue Incidence in Manaus, Amazonas, Brazil, 2016-2022

**DOI:** 10.1590/0037-8682-0325-2025

**Published:** 2026-07-17

**Authors:** Mirelia Rodrigues de Araujo, Francisco Chiaravalloti-Neto, Gerusa Maria Figueiredo

**Affiliations:** 1 Universidade de São Paulo, Faculdade de Medicina, Departamento de Medicina Preventiva, Programa de Pós-Graduação em Saúde Pública, São Paulo, SP, Brasil.; 2 Universidade de São Paulo, Escola de Saúde Pública, Departamento de Epidemiologia, São Paulo, SP, Brasil.; 3 Universidade de São Paulo, Faculdade de Medicina, Instituto de Medicina Tropical, Departamento de Medicina Preventiva, São Paulo, SP, Brasil.

**Keywords:** Dengue, Environment, Epidemiology, Spatiotemporal analysis

## Abstract

**Background:**

Dengue remains a public health challenge, driven by *Aedes aegypti* proliferation within complex socio-environmental conditions.

**Methods:**

This study investigated the spatiotemporal distribution of dengue in Manaus from 2016 to 2022. Temporal analysis used negative binomial regression adjusted for seasonality. Seasonal and spatial clusters were identified using scan statistics (p < 0.05, 95% CI). A Bayesian spatial model assessed sociodemographic and environmental determinants.

**Results:**

Lagged temperature and wind speed showed protective effects. The Bayesian model identified significant associations with population density, vegetation indices (NDVI), sewage infrastructure, and housing types.

**Conclusions:**

Integrating climatic and socioeconomic data is essential for intersectoral dengue control strategies.

Dengue activity has increased substantially in Latin America in recent years. Between 2023 and 2024, the epidemiological burden intensified, with Brazil accounting for most reported cases, which increase from 3,064,739 to 7,253,599 and represented 83.4% of the regional total in 2024^1^. 

Transmission dynamics are shaped by a complex interplay of climatic and anthropogenic factors. Seasonal patterns are strongly influenced by high temperatures and intense precipitation, which increase vector population density, shorten the extrinsic incubation period (EIP) of the dengue virus in mosquitoes, and enhance viral replication rates^2^ ^,^ ^3^. In addition, large-scale climate phenomena, such as El Niño and La Niña modulate seasonal precipitation in the Amazon region. In this context, El Niño years, associated with reduced rainfall and prolonged dry periods, tended to present lower dengue incidence because of reduced breeding-site availability, whereas La Niña years, characterized by increased precipitation, were associated with higher case numbers, partly due to more frequent flooding and water accumulation in urban areas^4^. 

Manaus, the capital of Amazonas state, has experienced recurrent dengue outbreaks since the 1990s, with sustained transmission and co-circulation of all four dengue virus serotypes, resulting in marked temporal variability. In this epidemiological context, this study examines the association between sociodemographic and urban environmental determinants and the spatial heterogeneity of dengue transmission within the city.

This ecological and analytical study was conducted in the urban area of Manaus, between 2016 and 2022, using census tracts as the spatial units of analysis. Four categories of variables were included: sociodemographic, climatological, environmental, and epidemiological variables, the latter represented by confirmed dengue cases.

Confirmed dengue cases were obtained from the Notifiable Diseases Information System (SINAN), provided by the Municipal Health Department of Manaus (SEMSA), and included demographic and residential information. Sociodemographic variables were derived from the National Registry of Addresses for Statistical Purposes (CNEFE) and the 2022 Brazilian Census (IBGE), encompassing population structure, housing conditions, access to basic sanitation, educational and health facilities, and population distribution by race/ethnicity. Environmental conditions were characterized using the mean Normalized Difference Vegetation Index (NDVI), calculated from Sentinel-2 imagery in Google Earth Engine (GEE) and interpreted according to standard criteria^5^. Climatic variables, including precipitation, temperature, humidity, wind speed, and number of rainy days, were obtained from the National Institute of Meteorology (INMET) and complemented with satellite-based data from CHELSA v2.1.

Temporal associations between dengue incidence and each climatic variable were assessed using separate negative binomial regression models. Seasonality was controlled by including monthly dummy variables as fixed effects, and lag structures of zero to three months were evaluated; results were expressed as incidence rate ratios (IRR) with 95% confidence intervals (p < 0.05). Seasonal and spatial clusters were identified using scan statistics with a discrete Poisson model in SaTScan, based on 999 Monte Carlo replications, with cluster size limited to ≤3% of the population to ensure non-overlapping clusters. After geocoding and address validation, annual dengue incidence rates per 100,000 inhabitants were calculated by census tract using 2022 IBGE population data, with rate stabilization applied to sparsely populated tracts. Associations between dengue risk and sociodemographic and environmental indicators were evaluated using a Bayesian spatial Poisson model with a Besag-York-Mollié structure fitted via Integrated Nested Laplace Approximation (INLA), and were expressed as relative risks with 95% credible intervals (CrI). Temporal and spatial analyses were conducted separately to improve interpretability of climatic and spatial effects. Different datasets were used for each analysis, and spatial modeling was based on a shorter period due to geocoding limitations.

Between 2016 and 2022, 4,540 confirmed dengue cases were recorded in Manaus. The highest incidence occurred among individuals aged 10-14 years (532.8/100,000), followed by those aged 15-19 years (277.4/100,000). Regarding educational level, 18.5% of cases occurred among individuals with incomplete or complete secondary education, although approximately 46.5% of records had missing information. Most cases were reported among individuals self-identified as mixed-race (89.4%). Clinical outcomes showed that 97.6% of cases resulted in recovery.

Monthly temporal analysis identified an average of 54 cases per month, with a peak of 512 cases. The highest incidence occurred from January to June, particularly in March and April (Figure 1), characterizing a seasonal cluster (RR=3.8; p=0.001).

**FIGURE 1:**
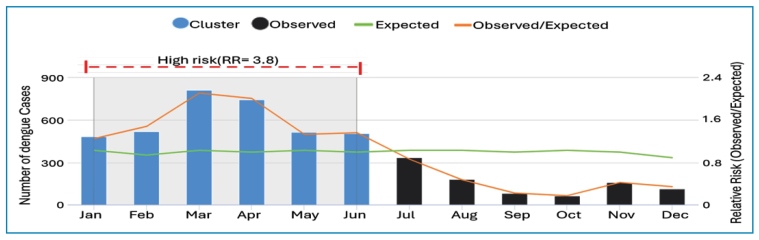
Seasonal distribution of observed and expected dengue cases and relative risk (Observed/Expected) in statistically significant clusters, urban area of Manaus, Brazil, 2016-2022. * Observed/Expected values correspond to the relative risk estimated by the Poisson model.

In the temporal regression model (Table 1), temperature was negatively associated with the number of dengue cases. Each 1°C increase in mean temperature reduced dengue risk by 38% (IRR=0.62; p=0.004). With a two-month lag, the reduction reached 61% (IRR=0.39; p<0.001). Maximum temperature also showed a significant protective effect (IRR=0.51; p<0.001), while mean wind speed with a two-month lag was associated with an 84% reduction in risk (IRR=0.16; p=0.019).

**TABLE 1:** Association between predictors and dengue incidence

**Variable**	No lag		Lag 1 month		Lag 2 month		Lag 3 month	
	IRR (%)	***p*-value (CI)**	IRR (%)	***p*-vaue (CI)**	IRR (%)	***p*-value (CI)**	IRR (%)	***p*-value (CI)**
Mean temperature	0.62 (38)	0.004 (0.45; 0.86)	0.51 (49)	<0.001 (0.35; 0.74)	0.39 (61)	<0.001 (0.27; 0.57)	0.41 (59)	< 0.001 (0.28;0.61)
Maximum temperature	-	-	0.60 (40)	0.001 (0.44; 0.82)	0.51 (49)	<0.001 (0.37; 0.69)	0.51 (49)	< 0.001 (0.37;0.70)

*p*-value= 0.005. **IRR:** Incidence Risk Ratios. **CI 95%:** Confidence Intervals 95%.

Of the addresses analyzed, 3,635 (95%) were successfully geocoded. A substantial proportion of addresses contained inconsistencies, which were resolved through data recovery and validation procedures. Twenty spatial clusters were identified, with the highest relative risks observed in the Center-West and West zones (RR range, 2.43-3.77; p<0.001). Low-risk clusters were mainly found in the South, North, and East zones, with the most prominent low-risk cluster located in the East zone (RR=0.54; p=0.01), covering 3.93 km² (Supplementary Figure 1).

In the spatial modeling (Table 2), the model including the proportion of mixed-race population performed better (DIC=7734.001; WAIC=7752.598) than the model including the proportion of white population. In the better-performing model, increased dengue risk was associated with the proportion of mixed-race residents, the proportion of buildings classified as houses (RR=2.19; 95% CrI: 1.69-2.79), the proportion of households with adequate sanitation (RR=1.39; CrI: 1.18-1.63), and the square root of the proportion of black population (RR=2.81; CrI: 1.06-6.09). Conversely, protective effects were observed for NDVI (RR=0.46; CrI: 0.25-0.78), population density (RR=0.95; CrI: 0.93-0.97), the proportion of permanent private households (RR=0.20; CrI: 0.11-0.35), and the presence of alleys or streams (RR=0.54; CrI: 0.37-0.78).

**TABLE 2:** Posterior means of Relative Risks (RRs), risk value as a percentage (%) and significant 95% Credible Intervals (CrI’s) from the Bayesian spatial model.

Variable	RR	%	CrI 95%	
(Intercept)	0.32	68	0.05	1.05
**rz.**_dens_	**0.55**	45	0.93	0.97
**Pr**_dom_par_	**0.20**	80	0.11	0.35
**Pr**_dom_cas_	**2.19**	119	1.69	2.79
**Pr**_Lbec_ig_	**0.54**	46	0.37	0.78
**Pr**_esgo_adq_	**1.39**	39	1.18	1.63
**rz.**_pr.Pret_	**2.81**	131	1.06	6.09
**NDVI**_mean_	**0.46**	54	0.25	0.78

* *Covariates whose credible intervals do not include the reference value are highlighted in bold.*

** Rz.dens: Population density ratio; **Prdom_par:** Proportion of private households; **rz.pr.constr:** Proportion of households under construction or renovation; **Prdom_cas:** Proportion of households classified as houses; **PrLbec_ig:** Proportion of streets/roadways classified as alleys or igarapes; **Prcons_adq:** Proportion of households supplied by the public water system plus deep or artesian wells; **Presgo_adq:** Proportion of households with sewage disposal via public or storm sewer system plus septic tank or filter-connected septic system; **Prabas_adq:** Proportion of households with piped water; **Prlixo_adq:** Proportion of households with waste collected by public cleaning services; **plus:** communal dumpsters; **Prpard:** Proportion of population identifying as Brown (Pardo); **Rz.Pr.Pret:** Ratio of the proportion of population identifying as black; **Rz.Pr.indig:** Ratio of the proportion of population identifying as Indigenous; **PrespFEM:** Proportion of households with female headship (female household responsibility); **NDVImean:** Mean Normalized Difference Vegetation Index; **Saude.ft / Ensino.ft1:** Health or education facilities; **Alf.cat.ft2.70a80:** Proportion of literate individuals aged 70-80; **Alf.cat.ft3.maior80:** Proportion of literate individuals aged over 80.

Relative risks (RR) for dengue were estimated using two Bayesian spatial models: an intercept-only and a covariate-adjusted model **(** Suplementary Figure 2 **)**. The intercept-only model revealed substantial spatial heterogeneity, with RR values ranging from 2.0 to 11.27, primarily concentrated in the Center-West zone and specific areas of the West zone, particularly near the Negro River and the surroundings of the Rio Negro Bridge. After covariate adjustment, the number of census tracts classified as high risk (RR, 1.1-11.27) increased, with persistent concentration in the Center-West and West zones. Simultaneously, the number of low-risk tracts (RR, 0.03-0.3) also increased, totaling 378 tracts, mainly located in the East and North zones.

Descriptive analysis showed that the highest incidence occurred among individuals aged 10-14 years. Over time, adults tend to develop broader immunity against dengue virus serotypes. This dynamic became evident following the reintroduction of DENV-1 in 1986, DENV-2 in 1990, and DENV-3 in 2002 in Brazil. As different serotypes circulate simultaneously for longer periods nationwide, the likelihood of adults remaining susceptible decreases^6^.

The climatic variables mean and maximum temperatures showed protective effects, consistent with evidence that higher temperatures may reduce mosquito survival despite generally favoring viral replication and vector development. Dengue transmission tends to peak within intermediate temperature ranges, and natural temperature fluctuations and regimes warrant further investigation to better understand the environmental effects on vectors and viruses^3^.

Mean wind speed was also negatively associated with dengue incidence, a protective effect that may reflect interference with the mosquito’s ability to detect hosts through skin odors^7^. A clear seasonal pattern was observed in Manaus, with higher dengue incidence from January to March. This seasonality has been associated with the El Niño-Southern Oscillation (ENSO), a well-known modulator of regional climate variability. However, in the context of climate change, ENSO characteristics appear to be changing, and ongoing studies seek to identify the factors regulating its frequency and magnitude^8^.

High-risk spatial clusters were concentrated in the Center-West and West zones, particularly near the Rio Negro Bridge, areas characterized by intense economic activity and urban connectivity. These environments may facilitate dengue transmission through increased population density, mobility, and contact rates^9^. 

Some statistically significant covariates were associated with increased dengue risk. The proportion of individuals identifying as black or mixed-race was linked to a 131% higher risk of infection. Although racial classification is based on self-identification and may be underreported, it remains an important proxy for socioeconomic disadvantage, which influences dengue risk through housing conditions, education, income, and access to healthcare^10^. The covariate representing adequate sanitation also showed a 39% positive association with dengue incidence. This apparently paradoxical result may reflect that, although sanitation improvements reduce risk at the household level, urban water and sewage infrastructure can unintentionally create favorable conditions for Aedes breeding, such as drainage systems and water-holding structures. Alternatively, this association may indicate improved socioeconomic conditions and healthcare access, leading to increased case detection rather than higher transmission^11^.

NDVI and population density were negatively associated with dengue incidence, by 54% and 45%, respectively. Areas with greater green-space coverage tend to exhibit lower population density because they are typically less urbanized, which may limit dengue transmission by reducing the absolute number of susceptible individuals and the intensity of human-vector contact^5^. Conversely, high population density was also associated with a lower relative risk of dengue, possibly reflecting an indirect effect of acquired immunity. In densely populated urban centers, sustained viral circulation over several decades may have produced greater accumulation of multitypic immunity, particularly among adults, thereby reducing the proportion of susceptible individuals. This pattern is consistent with the history of reemergence and co-circulation of the four dengue serotypes in Brazil and its various regions^6^.

Private households showed an 80% protective effect. This may relate to the intradomiciliary context, in which household composition influences infection risk, for example through protective window screens. Immunity again emerges as a potential pathway: each additional adult may reduce infection risk among residents, depending on prior infection history and the number of immune individuals in the household^12^. Alleyways and streams also showed negative associations with dengue incidence. In alleyways, artificial shading may inhibit oviposition because female Aedes generally prefer sunny environments^13^. In streams, waste collection and eco-barrier installations may reduce breeding site availability^14^. 

After covariate adjustment, spatial heterogeneity in dengue risk was reduced, suggesting that combined socioenvironmental factors exert a protective effect. Interactions between climatic and social determinants likely shape dengue transmission in complex, non-additive ways^15^, contributing to the persistent challenge of dengue control in Brazil.

This study highlights the spatiotemporal heterogeneity of dengue risk in Manaus and the role of climatic, environmental, and socioeconomic determinants. Although high incidence does not necessarily correspond to a high absolute number of cases, as incidence is influenced by population size, identifying high-risk areas remains essential. Some high-incidence areas may have smaller populations, whereas areas with lower incidence but larger populations may contribute more substantially to the overall dengue burden in the city.

This study has some limitations, including the relatively short time series and the absence of variables such as age, sex, urban mobility, vector control activities, and flooding intensity, which may have influenced the observed associations. Climatic variables were analyzed using individual models rather than a simultaneous multivariable approach due to potential collinearity between predictors such as temperature and rainfall. Although this strategy does not isolate independent effects, it allows clearer assessment of seasonal associations in Manaus. Future studies using longer time series and approaches such as distributed lag nonlinear models may further refine risk assessment.

This study was approved by the Research Ethics Committee of the Hospital das Clínicas, School of Medicine, University of São Paulo (Approval No. 5.673.365). 

## Figures and Tables

**SUPPLEMENTARY FIGURE 1:**
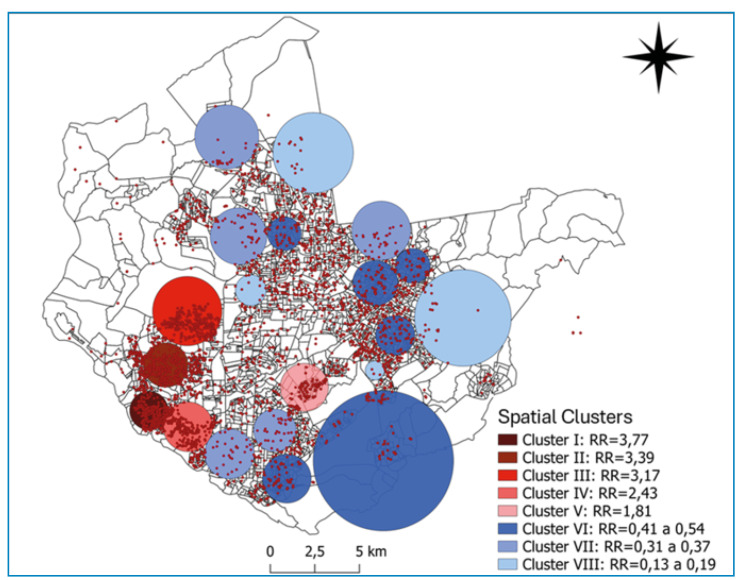
Purely spatial clusters (2018-2022). *RR: Relative Risk

**SUPPLEMENTARY FIGURE 2:**
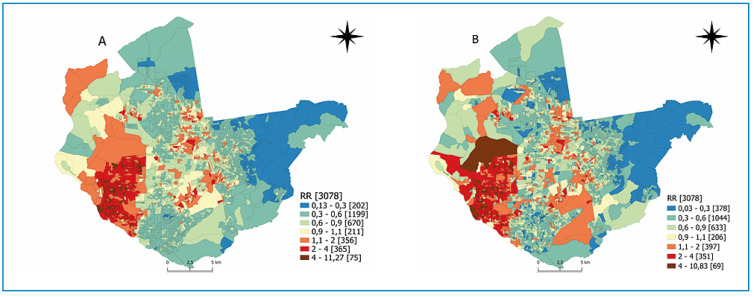
Maps of the posterior means of relative risks (RR) for the intercept-only model **(A)** and final model with covariates **(B)** in the urban area of Manaus. *RR: Relative Risk.

## Data Availability

upon-request.
